# Efficacy of ceftazidime/avibactam versus other antimicrobial agents for treating multidrug- resistant *Pseudomonas aeruginosa*: a propensity-matched retrospective analysis

**DOI:** 10.3389/fcimb.2025.1644991

**Published:** 2025-12-11

**Authors:** Qian Qian, JiaChen Wei, Fei Xu, XinYu Qin, Pei Ji, ChuWei Jing, ShuMei Miao, WenKui Sun

**Affiliations:** 1 Department of Clinical Medicine, Jiangsu Health Vocational College, Nanjing, Jiangsu, China; 2 Department of Respiratory and Critical Care Medicine, The First Affiliated Hospital with Nanjing Medical University, Nanjing, Jiangsu, China; 3 Department of Gastroenterology, The Second Affiliated Hospital with Nanjing Medical University, Nanjing, Jiangsu, China; 4 Department of Information, The First Affiliated Hospital with Nanjing Medical University, Nanjing, Jiangsu, China; 5 Department of Medical Informatics, School of Biomedical Engineering and Informatics, Nanjing Medical University, Nanjing, Jiangsu, China

**Keywords:** multidrug-resistant *Pseudomonas aeruginosa*, ceftazidime avibactam, propensity score-matched, clinical efficacy, microbiological efficacy, pulmonary infection

## Abstract

**Introduction:**

Multidrug-resistant *Pseudomonas aeruginosa* (MDRPA) is a life-threatening infection with limited treatment options. As a novel combination drug of cephalosporin and beta-lactamase inhibitor, ceftazidime/avibactam (CAZ/AVI) is not much used in clinical treatment of MDRPA infections. To fill in this knowledge gap, a single-center real-world study was conducted.

**Methods:**

This single-center retrospective observational study included MDRPA-infected patients treated with CAZ/AVI or other antimicrobial agents between January 2019 and April 2021. Propensity score-matched and binary logistic regression analysis was used to compare the clinical and microbiological efficacy between CAZ/AVI and other antimicrobial agents.

**Results:**

Totally 363 patients with MDRPA infection were enrolled, including 49 patients treated with CAZ/AVI and 314 patients treated with other antimicrobial agents. The CAZ/AVI group exhibited a reduced failure rate of clinical treatment (P = 0.012, OR = 0.381, 95% CI 0.180 - 0.807). Subgroup analysis showed single lung infection was a significant risk factor for clinical treatment failure in patients treated with other antimicrobial agents (P = 0.023, OR = 2.568, 95% CI 1.138 - 5.796). No significant discrepancy was observed in microbiological efficacy between the two groups (P = 0.159, OR = 0.587, 95% CI 0.280 - 1.232), or in clinical and microbiological efficacy between CAZ/AVI monotherapy and combination therapy.

**Conclusions:**

CAZ/AVI demonstrates superior clinical efficacy against MDRPA in comparison to other antimicrobial agents. However, the administration of CAZ/AVI as part of combination therapy does not provide any clear benefits over monotherapy. Patients with single pulmonary infection caused by MDRPA show better clinical efficacy with CAZ/AVI. Further larger studies are needed to substantiate our findings.

## 1 Introduction

Antimicrobial resistance is one of the most crucial public health challenges of the 21st century, and is largely attributed to multidrug-resistant Gram-negative bacteria (MDR-GNBs). The prevalence of MDR-GNB infections has increased alarmingly in recent decades (Morris and Cerceo, 2020). Concurrently, MDR-GNBs have become a leading cause of hospital-associated infections (Raman et al., 2018). Worryingly, the prevalence of carbapenemase production in carbapenem-resistant *Pseudomonas aeruginosa* (CRPA) in China has reached 41% (Zhang et al., 2023). Multidrug-resistant *Pseudomonas aeruginosa* (MDRPA) or CRPA is one major pathogen in the hospital setting, and is particularly challenging due to limited therapeutic options and high mortality rates (Zhang et al., 2016; Wei et al., 2020). Mortality from bloodstream infections caused by difficult-to-treat *Pseudomonas aeruginosa* (DTR-PA) is up to 50% (Yuan et al., 2023). Global rates of carbapenem resistance in *P. aeruginosa* generally range from 10% to 20% and MDR rates vary from 5% to 30%, depending on the region and the site of infection (Macesic et al., 2025).

Ceftazidime/avibactam (CAZ/AVI) is a combination of an anti-pseudomonal cephalosporin and a novel non-β-lactam β-lactamase inhibitor. It is now available for treatment of MDR-GNB infections, and has brought favorable outcomes to hospitalized patients with carbapenem-resistant Enterobacterales (CRE) (Chen et al., 2021; Almangour et al., 2022; Castón et al., 2022). However, only a few publications with small sample sizes and focusing on the efficacy of CAZ/AVI for treatment of CRPA or MDRPA infections are available. Therefore, a propensity-matched retrospective analysis was conducted to compare the clinical efficacy and microbiological efficacy of CAZ/AVI versus other antimicrobial agents for treatment of MDRPA infections.

## 2 Materials and methods

### 2.1 Study setting and patient selection

This single-center, retrospective and observational study was conducted in the First Affiliated Hospital of Nanjing Medical University, a 2200-bed tertiary care teaching hospital in Nanjing, Jiangsu in China, from January 2019 to April 2021. Patients were included if they met the following criteria: (1) MDRPA infection diagnosed by clinicians, (2) treatment with anti-*P. aeruginosa* antibiotics for at least 72 h, and (3) older than 18 years old. Exclusion criteria comprised: (1) patients with human immunodeffciency virus infection, malignant tumors undergoing radiotherapy and chemotherapy, high-dose hormone therapy, immunosuppressive therapy, receipt of bone marrow or solid organ transplant and other immunosuppressive states; (2) mental illness; (3) antimicrobial therapy duration <72 hours; or (4) incomplete clinical data. To further investigate the efficacy of CAZ/AVI compared with other antimicrobial agents in treatment of *P. aeruginosa* infections, the population was divided into a CAZ/AVI treatment group and a control group (other antimicrobial agents) according to whether or not CAZ/AVI was used for treatment. This study was conducted in accordance with *the Declaration of Helsinki*, and approved by the Institutional Review Committee of the First Affiliated Hospital with Nanjing Medical University (2021-SR-446). A large amount of data was automatically collected and generated through the database to control selection bias and information bias related to the study.

### 2.2 Data collection

Patients’ information was obtained from electronic medical records. The baseline data consisted of age, gender, BMI, whether the airway was open, treatment course, status of combined use of other antimicrobial agents, and whether the patient was admitted to the ICU, peak temperature at the time of infection, leukocyte counts, neutrophil percentage, procalcitonin (PCT) levels, and status of bacterial cultures after the administration of medication.

### 2.3 Definitions

The definition of pulmonary infection was based on the Infectious Diseases Society standard (Miller et al., 2018). MDR bacteria were considered to have acquired non-susceptibility to at least one agent in three or more antimicrobial categories (Magiorakos et al., 2012). A diagnosis of MDRPA pulmonary infection required congruent clinical signs plus the isolation of MDRPA from a qualified sputum or BALF specimen, defined by ≥25 neutrophils and <10 epithelial cells per low-power field and at least moderate bacterial growth on semiquantitative culture (Fujitani et al., 2009). The definition of urinary tract infection was based on the European Association of Urology guidelines (Kranz et al., 2024), requiring a combination of clinical symptoms (such as dysuria, frequency, or suprapubic pain) and significant bacteriuria (≥10³ CFU/mL in a midstream urine sample). The definition of intra-abdominal infection was based on the The Surgical Infection Society guidelines on the management of intra-abdominal infection (Huston et al., 2024), encompassing infections involving the intra-abdominal organs or spaces, evidenced by clinical signs (e.g., abdominal pain, tenderness), and/or radiological findings, with or without microbiological confirmation. The definition of bloodstream infection was based on guidelines released by the Infectious Diseases Society of America (Liu et al., 2011), defined by the presence of viable bacteria or fungi in the bloodstream, confirmed by one or more positive blood cultures, and accompanied by systemic signs of infection (e.g., fever, chills, hypotension).

### 2.4 Study assessments

Two senior physicians with extensive clinical experience classified the clinical efficacy of the treatment as effective (characterized by the resolution or significant improvement of clinical symptoms and signs, normalization or minimal residuals in pulmonary imaging, and normalization of inflammatory markers), uncertain or ineffective (Torres et al., 2018). Microbiological efficacy was defined as eradication or reduction, indicated by the absence of MDRPA in lower respiratory tract secretion cultures after treatment, or a decrease in bacterial load by more than 25% compared to pre-treatment levels (Wang et al., 2022). Microbiological ineffectiveness was defined as the continued presence of MDRAB in cultures after treatment and no change or an increase in bacterial load compared to pre-treatment levels (Wang et al., 2022).

### 2.5 Statistical analysis

Categorical variables were expressed as absolute numbers and relative frequencies (%), and compared using Chi-square test or Fisher’s exact test. Continuous variables were expressed as mean ± standard deviation and compared using t-test when they followed a normal distribution. Continuous variables not in normal distribution were expressed as median and interquartile range (IQR), and compared with Mann-Whitney U-test. Treatment efficacy between the two groups was compared using logistic regression analysis. A one-to-three propensity score matching (PSM) was adopted with a caliper width of 0.2. Variables adjusted for PSM in the study included: males, age, BMI, temperature, ICU admission, open airway, pulmonary infection, co-infection with other bacteria, co-infection with *Acinetobacter baumannii*, co-infection with *Klebsiella pneumoniae*, leukocyte and procalcitonin. All statistical analyses were performed in IBM SPSS Statistics 26.0, and P values < 0.05 were significant.

## 3 Results

### 3.1 Characteristics of patients

**Figure 1:**
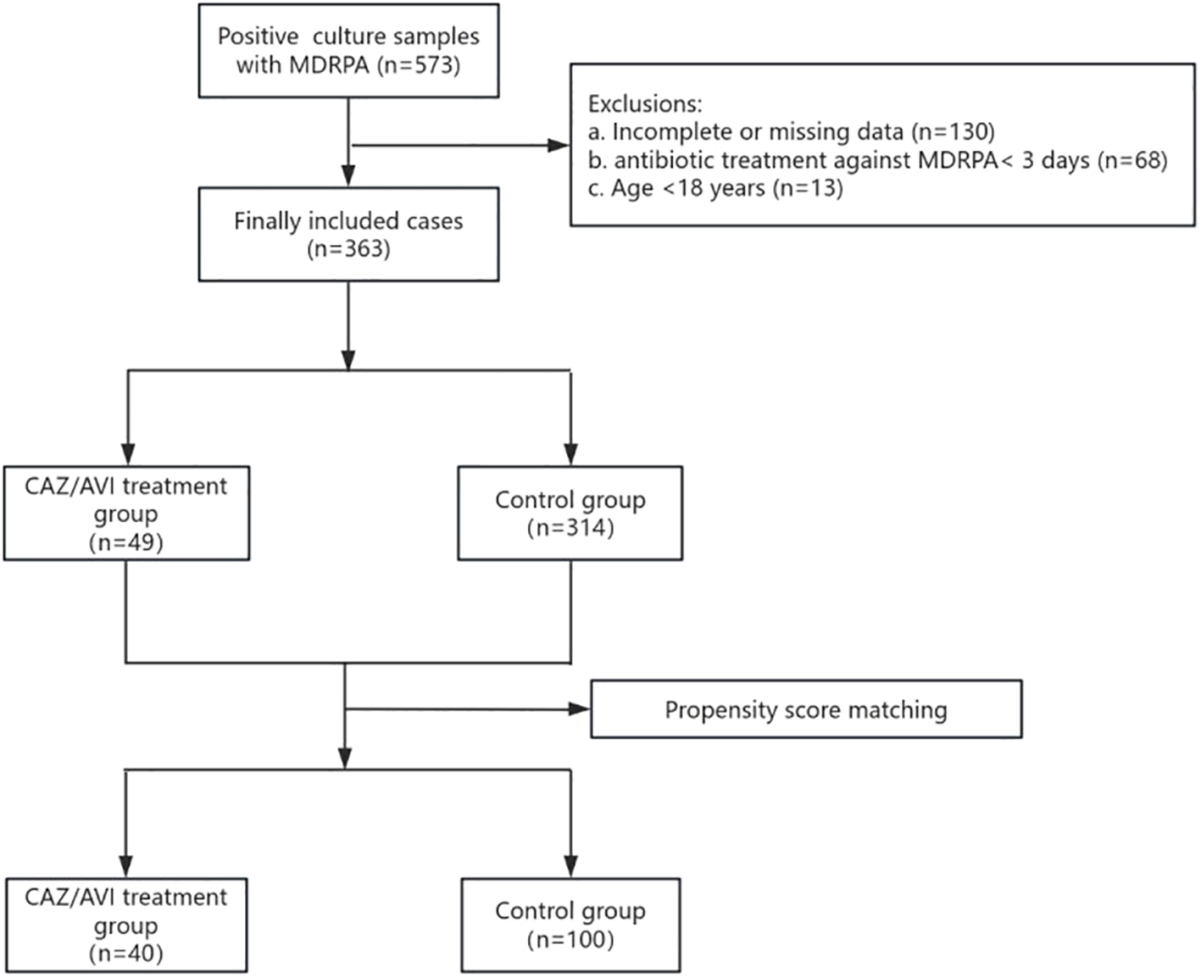
Flowchart of the patients included in the study. MDRPA, Multidrug-resistant Pseudomonas aeruginosa. CAZ/AVI, ceftazidime/avibactam.

Of the 573 cases with positive cultures for MDRPA, 363 episodes met the inclusion criteria and were included in the final analysis (**Figure 1**). Among them, 49 patients were treated with CAZ/AVI, while 314 patients were treated with other antimicrobial agents. Of these patients, 66.67% (n = 242) were male, and 52.62% (n = 191) were admitted to the ICU. The proportion of pulmonary infections was 91.74%. About 27.27% of the patients were infected with other pathogens, primarily *Acinetobacter baumannii* (10.74%) and *Klebsiella pneumoniae* (9.92%).

### 3.2 Baseline characteristics of unmatched patients

In comparison with the control group, the CAZ/AVI treatment group demonstrated higher levels of peak temperature, leukocyte counts and procalcitonin. Males, ICU admission, open airway, co-infection with other bacteria and co-infection with *Klebsiella pneumoniae* were statistically more prevalent in the CAZ/AVI treatment group, while pulmonary infection was more frequently observed in the other antimicrobial agents treatment group. No significant differences were observed in other baseline characteristics (**Table 1**). The specific medication plans for each group are detailed in **Supplementary Table 1**.

**Table 1:** Baseline characteristics of unmatched patients.

Characteristic	CAZ/AVI treatment group (n = 49)	Control group (n = 314)	t/χ2/*Z*	P
Male	40 (81.6)	202 (64.3)	5.709	0.017
Age	65.02 ± 20.68	63.24 ± 16.03	0.578	0.488
BMI	23.95 ± 4.23	23.24 ± 4.65	1.011	0.313
Temperature	39.16 ± 0.84	38.23 ± 1.01	6.908	<0.001
ICU admission	39 (79.6)	152 (48.4)	16.533	<0.001
Open airway	40 (81.6)	196 (62.4)	6.878	0.009
Pulmonary infection	38 (77.6)	295 (93.9)	12.826	<0.001
Co- infection with other bacteria	23 (46.9)	76 (24.2)	11.045	0.001
Co-infection with *Acinetobacter baumannii*	9 (18.4)	30 (9.6)	3.433	0.064
Co-infection with *Klebsiella pneumoniae*	12 (24.5)	24 (7.6)	13.465	<0.001
Leukocyte	10.05 (7.34 - 14.24)	7.98 (5.76 - 11.85)	-2.873	0.004
PCT	1.24 (0.47 - 8.83)	0.30 (0.10 - 1.12)	-5.149	<0.001

### 3.3 Baseline characteristics of matched patients

Following the implementation of a PSM process, 140 patients were matched, with 40 patients allocated to the CAZ/AVI treatment group and 100 patients allocated to the control group. The baseline characteristics of the treatment groups following the application of the propensity score matching process are presented in **Table 2**. No significant differences were observed in the baseline characteristics of the treatment groups following the implementation of the propensity score matching process, apart from procalcitonin (p = 0.012).

**Table 2:** Baseline characteristics of matched patients.

Characteristic	CAZ/AVI treatment group (n = 40)	Control group (n = 100)	t/χ2/Z	P
Male	31 (77.5)	77 (77.0)	0.004	0.949
Age	67.38 ± 20.94	64.25 ± 14.55	0.864	0.391
BMI	24.00 ± 4.15	23.58 ± 5.32	0.452	0.652
Temperature	38.97 ± 0.78	38.96 ± 0.74	0.028	0.977
ICU admission	32(80.0)	73 (73.0)	0.747	0.388
Open airway	32 (80.0)	81 (81.0)	0.018	0.892
Pulmonary infection	34 (85.0)	93 (93.0)	2.171	0.141
Co- infection with other bacteria	17 (42.5)	30 (30.0)	2.002	0.157
Co-infection with *Acinetobacter baumannii*	7 (17.5)	14 (14.0)	0.275	0.600
Co-infection with *Klebsiella pneumoniae*	7 (17.5)	14 (14.0)	0.275	0.600
Leukocyte	10.03 (7.11 - 14.33)	9.93 (6.89 - 13.35)	-0.221	0.825
PCT	0.99 (0.44 - 7.42)	0.52 (0.20 - 1.68)	-2.505	0.012

### 3.4 Therapeutic effect comparison

Following the elimination of confounding factors, an analysis was conducted to explore the correlation between the use of CAZ/AVI (i.e., the CAZ/AVI treatment group) or its non-use (i.e., the control group) and clinical and microbiological treatment failure. The findings demonstrated that the CAZ/AVI group exhibited a reduced failure rate of clinical treatment (P = 0.012, OR = 0.381, 95% CI 0.180 - 0.807). Additionally, no statistically significant discrepancy was observed in terms of microbiological efficacy between the two groups (P = 0.159, OR = 0.587, 95% CI 0.280 - 1.232) (**Table 3**).

**Table 3:** Correlation analysis between the use of CAZ/AVI and clinical and microbiological efficacy.

The use of CAZ/AVI	B	P	OR	95% CI
Clinical efficacy	-0.966	0.012	0.381	0.180 - 0.807
Microbiological efficacy	-0.532	0.159	0.587	0.280 - 1.232

### 3.5 Efficacy analysis of CAZ/AVI monotherapy and combination therapy

Among the 49 patients treated with CAZ/AVI, 33 patients were treated with CAZ/AVI monotherapy during MDRPA infection, and 16 patients were treated with CAZ/AVI combined with other antimicrobial agents (including polymyxins and quinolones,etc.). The analysis demonstrated that there was no significant difference in clinical and microbiological efficacy between CAZ/AVI monotherapy or combination therapy (**Table 4**).

**Table 4:** Efficacy analysis of CAZ/AVI monotherapy and combination therapy.

Treatment regimen	Clinical success	Clinical failure	χ2	P	Microbiological success	Microbiological failure	χ2	P
Monotherapy therapy	19 (57.6)	14 (42.4)	0.567	0.452	15 (45.5)	18 (54.5)	2.348	0.125
Combination therapy	11 (68.8)	5 (31.3)			11 (68.8)	5 (31.3)		

### 3.6 Subgroup analysis by sources of infection

Binary logistic regression analysis was used to analyze the correlation between different infection sources and clinical and microbiological treatment failure. This study included 322 cases of single lung infections and 28 cases of other single source infections, including 18 cases of urinary tract infections, 7 cases of intra-abdominal infections, and 3 cases of bloodstream infections. 13 cases of other mixed source infections were identified, including 8 cases of urinary tract infections in combination with pulmonary infections, 3 cases of intra-abdominal infections in combination with pulmonary infections, and 2 cases of intra-abdominal infections in combination with bloodstream infections. Due to the limited number of cases from other sources of infection, the study categorized the sources of infection into the following: single pulmonary infection, other single source infections, and mixed source infections. The analysis revealed that single lung infection was a significant risk factor for clinical treatment failure in the other antimicrobial agents treatment group (P = 0.023, OR = 2.568, 95% CI 1.138 - 5.796). The study further revealed that there was no significant difference in the microbiological efficacy of single lung infection between the two groups, nor was there a significant difference in the clinical and microbiological efficacy of other infection sources between the two groups (**Tables 5**, **6**).

**Table 5:** Subgroup analysis of clinical failure according to source of infection.

Clinical failure	CAZ/AVI treatment group (n = 49)	Control group (n = 314)	P	OR	95% CI
Single pulmonary infection	9/30 (30.0%)	153/292 (52.4%)	0.023	2.568	1.138 - 5.796
Other single source infections	5/10 (50.0%)	11/18 (61.1%)	0.570	1.571	0.330 - 7.481
Mixed source infections	5/9 (55.6%)	3/4 (75.0%)	0.512	2.400	0.175 - 32.879

**Table 6:** Subgroup analysis of microbiological failure according to source of infection.

Microbiological failure	CAZ/AVI treatment group (n = 49)	Control group (n = 314)	P	OR	95% CI
Single pulmonary infection	15/30 (50.0%)	153/292 (52.4%)	0.802	1.101	0.519 - 2.334
Other single source infections	5/10 (50.0%)	8/18 (44.4%)	0.778	0.800	0.170 - 3.767
Mixed sources infections	3/9 (33.3%)	3/4 (75.0%)	0.186	6.000	0.422 - 85.248

## 4 Discussion

*P. Aeruginosa* has long been regarded as the most concerning pathogens worldwide. It is ranked tenth among antibiotic-resistant bacteria in the 2024 WHO Bacterial Priority Pathogens List (BPPL) (WHO, 2024). In China, the most recent data from the CHINET surveillance system revealed that *P. aeruginosa* accounted for 7.4% of 458,271 bacterial isolates collected through active surveillance across 74 tertiary hospitals in China in 2024 (http://www.chinets.com). Data from the CHINET surveillance system in 2023 showed that *P. aeruginosa* was the fourth most common bacterium isolated from respiratory samples. A multinational observational study involving 3,540 ICU patients with Gram-negative infections demonstrated that 24% (n = 850) of cases were attributable to *P. aeruginosa*, with respiratory tract infections representing the predominant site (Vincent et al., 2020). Infections due to MDRPA constitute an emerging health problem. A retrospective cohort study showed that 30.5% patients (n = 226) of 740 patients with *P. aeruginosa* nosocomial pneumonia (Pa-NP) were infected with MDR strains. Among the patients with Pa-NP, the presence of infection with an MDR strain is associated with increased in-hospital mortality (Micek et al., 2015). It is an established fact that MDRPA strains frequently exhibit multiple antimicrobial resistance mechanisms, particularly extended-spectrum β-lactamases (ESBLs) and increasingly prevalent carbapenemases (Abdelaziz et al., 2024), severely limiting therapeutic options for these infections.

The first generation of β-lactamase inhibitors, encompassing clavulanate, sulbactam and tazobactam, primarily target class A enzymes and partially inhibit class C enzymes, though their activity against carbapenemases remains negligible (Sood, 2013). CAZ/AVI is a combination cephalosporin and beta-lactamase inhibitor that was approved by the U.S. Food and Drug Administration in February 2015. Avibactam belongs to a class of inhibitors called diazabicyclooctanes (Coleman, 2011). In comparison with other β-lactamase inhibitors, avibactam is a novel covalent, slowly reversible non-rsible,0 inhibitor (Ehmann et al., 2012), and can inhibit class A enzymes (including ESBLs and KPC-type carbapenemases), class C enzymes, and some class D enzymes (including OXA-48-type) (de Jonge et al., 2016; Bush and Bradford, 2019), but cannot inhibit the activity of metallo-β-lactamases (Zhanel et al., 2013; Torrens et al., 2016). KPC production has been identified as the most prevalent cause of carbapenem resistance in *K. pneumoniae* in China (Li et al., 2022). Some research indicates the superiority of CAZ/AVI over other regimes in treatment of infections caused by carbapenem-resistant *Klebsiella pneumoniae* (CRKP) (Gu et al., 2021; Karampatakis et al., 2023). Depending on the resistance mechanisms involved, CAZ/AVI could be the most effective treatment option for certain MDRPA strains, including those that produce class A carbapenemases (GES enzymes) or combinations of certain ESBLs with loss of the OprD porin (Horcajada et al., 2019).

The effectiveness of CAZ/AVI and other antimicrobial agents in treating *P. aeruginosa* infections was compared previously. The antimicrobial activity of CAZ/AVI was tested against 7,868 *P. aeruginosa* isolates collected from 94 hospitals in the USA. CAZ/AVI showed potent activity against *P. aeruginosa* (97.1% susceptible), including MDR (86.5% susceptible) isolates, and inhibited 71.8% of isolates that were not susceptible to meropenem, piperacillin-tazobactam and ceftazidime (n = 628) (Sader et al., 2017). In a phase III clinical trial program, CAZ/AVI demonstrated similar clinical efficacy to predominantly carbapenem comparators against MDRPA (85.4% vs. 87.9%) (Stone et al., 2018). Chen et al. enrolled 136 CRPA-infected patients in a single center in China to provide a comparison of CAZ/AVI and polymyxin (Chen et al., 2022). The 14-day mortality rate (5.9% vs. 27.1%, P = 0.002), 30-day mortality rate (13.7% vs. 47.1%, P < 0.001), and in-hospital rate (29.4% vs. 60.0%, P = 0.001) were lower in the CAZ/AVI group than in the polymyxin B group. A recently published systematic review and meta-analysis, which to our knowledge is the first and most comprehensive on this topic, provides a crucial synthesis of the existing evidence regarding CAZ/AVI for MDRPA infections (Gupta et al., 2024). The pooled results demonstrated that CAZ/AVI was associated with significantly lower mortality compared to other antimicrobial regimens. This high-level evidence strongly corroborates the superior clinical efficacy of CAZ/AVI observed in our propensity score-matched analysis. With regard to the bacterial eradication, the frequency was significantly higher in those treated with CAZ/AVI versus polymyxin B (45.1% vs. 12.9%, P < 0.001). Compared to our research, these researchers did not implement PSM to eliminate intergroup differences, which made interpretation difficult. No significant difference in microbiological efficacy was observed between the CAZ/AVI group and the control group (50.0% vs. 37.0%, P = 0.159) after PSM. However, the CAZ/AVI group was associated with higher clinical treatment efficacy compared to the control group (57.5% vs. 34%, P = 0.012). Given the small sample size of this study and the fact that some microbiological treatments were ineffective in patients, the load decreased (despite the continued presence of the pathogen), potentially resulting in an improvement in the patients’ clinical symptoms.

The role of CAZ/AVI combination therapy for CRE remains debated. A recent meta-analysis including 13 studies and 503 patients with CRE infections found no difference in mortality between CAZ/AVI monotherapy and CAZ/AVI-based combination therapy (OR 0.96, 95% CI 0.65,pytio (Fiore et al., 2020). Similarly, a large retrospective study involving 577 adults infected with KPC-producing *K. pneumoniae* showed no difference between CAZ/AVI monotherapy and combination therapy (26.1% vs. 25.0%, P = 0.790) (Tumbarello et al., 2021). However, a retrospective cohort performed at two tertiary hospitals in China first reported that 30-day mortality rates for patients receiving CAZ/AVI-based combination therapy were significantly lower than those with monotherapy (24.4% vs. 47.6%, P = 0.028) for CRKP infection (Zheng et al., 2021). Given the existence of confounders such as illness severity and types of infections, more evidence is required to explore the potential benefit of combination for CRE infections.

Nevertheless, research on the effects of CAZ/AVI monotherapy and CAZ/AVI-based combination therapy on *P. aeruginosa* remains scarce. A retrospective cohort study included 61 patients with MDR/XDR-PA infections who received CAZ/AVI treatment for a minimum of 24 hours (Corbella et al., 2022). The rates of clinical cure by day 14 were comparable between episodes in which CAZ-AVI was administered as monotherapy or combination therapy (62.5% vs. 44.8%, P = 0.167). Aligning with our findings, no significant difference in clinical efficacy (57.58% vs. 68.75%) or microbiological efficacy (45.45% vs. 68.75%) was identified between CAZ/AVI monotherapy and combination therapy for the treatment of MDRPA infections. These data suggest that routine CAZ/AVI combination therapy may not confer additional benefits for MDRPA infections.

We analyzed the correlation between different infection sources and clinical/microbiological treatment failure rates. The analysis demonstrates that CAZ/AVI is more efficacious than other antimicrobial agents in treating single pulmonary infections due to MDRPA (52.4% vs. 30.0%, P = 0.023). However, a phase III clinical trial including adults with nosocomial pneumonia (including ventilator-associated pneumonia) from 136 centers in 23 countries was conducted to assess the efficacy and safety of CAZ/AVI compared with meropenem (Gu et al., 2021). The clinical cure rates and microbiological eradication rates of patients with *P. aeruginosa* pulmonary infection were generally comparable between treatment groups. Given the limited sample size, further research with a larger sample size is necessary to provide stronger evidence to support these findings.

This study has several limitations. Firstly, in real-world clinical settings, not all patients were rechecked for MDRPA colonization (e.g., sputum culture) at the end of drug therapy, which may have impacted our assessment of efficacy. Additionally, due to the limited number of cases involving infection source other than pulmonary infections, we did not perform further subgroup analyses on urinary tract infections, intra-abdominal infections, or bloodstream infections. Lastly, in this retrospective and non-randomized study, the amount and duration of antibiotics between groups may have introduced bias. Future research employing prospective or matched-pair design is needed to evaluate the efficacy of CAZ/AVI in treating MDRPB infections.

## 5 Conclusions

CAZ/AVI demonstrates superior clinical efficacy against MDRPA in comparison to other antimicrobial agents. The administration of CAZ/AVI as part of combination therapy provides no clear benefits over monotherapy. Patients with single pulmonary infection caused by MDRPA show better clinical efficacy with CAZ/AVI. However, given the limited number of included patients, further prospective studies with larger samples are needed to compare CAZ/AVI with other antimicrobial agents for MDRPA infections and test the potential benefits derived from the use of CAZ/AVI-containing combination regimens.

## Data Availability

The original contributions presented in the study are included in the article/**Supplementary Material**. Further inquiries can be directed to the corresponding authors.
