# Supplementary material for: Efficacy of ceftazidime/avibactam versus other antimicrobial agents for treating multidrug- resistant Pseudomonas aeruginosa: a propensity-matched retrospective analysis
Source: Front Cell Infect Microbiol. 2025 Dec 11;15:1644991. doi: 10.3389/fcimb.2025.1644991 (PMC12738922; doi:10.3389/fcimb.2025.1644991)
Supplement: Supplementary file 1 [file DataSheet1.pdf]

Supplementary file

Supplementary Table 1 Medication plan of the 363 patients with MDRPA.

Antimicrobial agents	Number of patients n (%)
CAZ/AVI treatment group	
CAZ/AVI[1]	33(67.3%)
CAZ/AVI combined with polymyxins[1,2]	8(16.3%)
CAZ/AVI combined with quinolones[1]	6(12.2%)
CAZ/AVI combined with aminoglycosides[1]	2(4.1%)
Control group	
Carbapenems[2]	54(17.2%)
Other β-lactam/β-lactamase inhibitor combinations[3]	41(13.1%)
Other β-lactam/β-lactamase inhibitor combinations combined with polymyxins[3,4]	98(31.2%)
Other β-lactam/β-lactamase inhibitor combinations combined with quinolones[3]	65(20.7%)
Other β-lactam/β-lactamase inhibitor combinations combined with aminoglycosides[3]	18(5.7%)
Carbapenems combined with polymyxins[3,5]	27(8.6%)
Carbapenems combined with aminoglycosides[3]	11(3.5%)

[1]Percentage in relation to CAZ/AVI treatment group.

[2]The administration of polymyxins was via Intravenous infusion for 6 patients and via nebulisation for 2 patients.

[3]Percentage in relation to Control group.

[4]The administration of polymyxins was via Intravenous infusion for 86 patients and via nebulisation for 12 patients.

[5]The administration of polymyxins was via Intravenous infusion for 10 patients and via nebulisation for 1 patient.

Supplementary Table 2 Antimicrobial susceptibilities of MDRPA isolates to selected antimicrobial agents.

N (%)	CAZ/AVI	Colistin	Quinolone	aminoglycoside	Carbapenem	Cefoperazone/sulbactam	Piperacillin/tazobactam
Tested isolates[1]	86(23.7%)	52(14.3%)	363(100%)	363(100%)	363(100%)	111(30.6%)	363(100%)
Susceptible [2]	65(75.6%)	45(86.5%)	148(40.8%)	195(53.7%)	155(42.7%)	25(22.5%)	104(28.7%)
Non-susceptible [2]	21(24.4%)	7(13.5%)	215(59.2%)	168(46.3%)	208(57.3%)	86(77.5%)	259(71.3%)

[1]Percentage in relation to all isolates.

[2]Percentage in relation to the tested isolates.
